# The Recent Past and Possible Futures of Citizen Science: Final Remarks

**DOI:** 10.1007/978-3-030-58278-4_26

**Published:** 2020-08-29

**Authors:** Josep Perelló, Andrzej Klimczuk, Anne Land-Zandstra, Katrin Vohland, Katherin Wagenknecht, Claire Narraway, Rob Lemmens, Marisa Ponti

**Affiliations:** 1 Museum für Naturkunde Berlin – Leibniz, Institute for Evolution and Biodiversity Science (MfN), Berlin, Germany; 2 Faculty of Science, Leiden University, Leiden, The Netherlands; 3 Earthwatch Europe, Oxford, UK; 4 Faculty of Geo-Information Science and Earth Observation (ITC), University of Twente, Enschede, The Netherlands; 5 OpenSystems, Departament de Física de la Matèria Condensada, Universitat de Barcelona, Barcelona, Spain; 6 Department of Applied Information Technology, University of Gothenburg, Gothenburg, Sweden; 7 Department of Bioscience Engineering, University of Antwerp, Antwerp, Belgium; 8 Museum für Naturkunde Berlin – Leibniz, Institute for Evolution and Biodiversity Science (MfN), Berlin, Germany; 9 OpenSystems, Departament de Física de la Matèria Condensada, Universitat de Barcelona, Barcelona, Spain; 10 SGH Warsaw School of Economics, Warsaw, Poland; 11 Faculty of Science, Leiden University, Leiden, The Netherlands; 12 Museum für Naturkunde Berlin – Leibniz Institute for Evolution and Biodiversity Science (MfN), Berlin, Germany; 13 Natural History Museum (NHM), Vienna, Austria; 14 Earthwatch Europe, Oxford, UK; 15 Faculty of Geo-Information Science and Earth Observation (ITC), University of Twente, Enschede, The Netherlands; 16 Department of Applied Information Technology, University of Gothenburg, Gothenburg, Sweden

## Abstract

This book is the culmination of the COST Action CA15212 *Citizen Science to Promote Creativity, Scientific Literacy, and Innovation throughout Europe*. It represents the final stage of a shared journey taken over the last 4 years. During this relatively short period, our citizen science practices and perspectives have rapidly evolved. In this chapter we discuss what we have learnt about the recent past of citizen science and what we expect and hope for the future.

## The COST Action: The Recent Past

This book is the culmination of the COST Action^1^ CA15212 *Citizen Science to Promote Creativity, Scientific Literacy, and Innovation throughout Europe*. It represents the final stage of a shared journey taken over the last 4 years. During this relatively short period, our citizen science practices and perspectives have rapidly evolved.

The COST Action started in 2016, when citizen science was gaining momentum in Europe and worldwide. The first international citizen science conference took place in San José, California, in 2012. This period also saw the foundation of citizen science organisations, such as the European Citizen Science Association (ECSA) at the Museum für Naturkunde Berlin, in 2014. These milestones were not isolated events in the evolution of citizen science. There was a confluence of factors on multiple levels: globally, nationally, and locally. There was a sense of urgency to find common spaces to discuss the widespread flourishing of citizen science practices. These factors led to the formation of the citizen science COST Action.

The impetus for citizen science in Europe over the last few years is partially indebted to the activities and interactions of this COST Action. This has offered a panoramic view of new initiatives, recently built digital platforms, and ongoing hot topic debates in the citizen science community of practitioners. It also helped spark several European-funded projects. The most relevant example is EU-Citizen.Science, a coordination and support platform launched in 2019. Its goal is to become the European reference point for citizen science, through cross-network knowledge sharing on a multi-language repository website with access to projects and resources for all stakeholders.

Since 2016, the COST Action has expanded the network of people involved in citizen science practices in Europe. Even in its embryonic stage, the COST Action was a large-scale networking exercise, with the proposal writing being led by Claudia Göbel, Marisa Ponti, and Katrin Vohland. When the COST Action was launched, the initial community expanded rapidly to 500 participating individuals in 39 member countries. The success in terms of number of participants, however, meant that COST Action management and governance became more challenging than initially anticipated by the COST co-chairs, Katrin Vohland, Marisa Ponti, and Anne Land-Zandstra (who replaced Marisa Ponti when she started a new position at the EC Joint Research Centre). Reaching consensus was not always easy. Sometimes it was hard to get everyone on the same page or to engage them in the multiple issues that COST Actions face. For everyone, the COST Action activities involved a commitment beyond their organisational roles. The COST Action refunds travel costs to members, but it does not provide support with regard to, for instance, personal costs. It was therefore challenging for many of the stakeholders to invest time and energy in the COST Action. Co-chairs worked hard to balance the diverse interests of a large group of people and ensure all their efforts could be best aligned. As a COST Action citizen science community, we acknowledge the co-chairs for their dedicated time commitment.

The COST Action has been an arena for connecting with citizen science initiatives across Europe, from Greece to Ireland, from Norway to Spain. COST meetings have included many people from various countries, with diverse backgrounds, experiences, and expertise. It has broadened understanding of what citizen science looks like in different parts of Europe and across the world. The case of Central and Eastern Europe has been particularly interesting, since citizen science is in its infancy. It was somewhat hidden and generally initiated from different sociopolitical contexts, compared to other European countries. The COST Action has also strengthened the links between us, which will no doubt lead to continued collaboration in the future.

The COST Action has also offered workshops, short-term scientific missions, and training schools to share and exchange ideas. These have brought together a wide variety of viewpoints on citizen science and provided support to develop them. The COST Action also allowed us to learn about different aspects of citizen science from our peers in relation to terminologies, conceptualisations, and theoretical frameworks and also practical issues such as data management and interoperability. However, above all, it is always great to sit alongside others who are excited about the same things. This proximity has increased our self-confidence, self-esteem, and enthusiasm for citizen science practices.

The COST Action has enabled an academic forum to emerge for connecting disciplines and consolidating citizen science as a scientific practice. The COST Action has been a shared space, bypassing disciplinary differences, to enable the discussion of common transversal issues. For example, an economist and a public policy expert have found in the COST Action a space to work together with scholars from environmental science and the computational sciences. Computational sciences practitioners have indeed increased their presence as they are interested in shifting from a users’ paradigm to a participants’ paradigm, when referring to crowdsourcing and collective intelligence digital platforms. The computational sciences are expected to further increase their presence in the citizen science world in order to build better infrastructures to increase active citizenship, driving citizen science initiatives and embracing ethical and legal issues.

Many scholars from the natural sciences have also learnt from social scientists. Social scientists are increasingly needed to improve citizen science practices in terms of fair citizen participation and project research goals. Social scientists can contribute to improved reflection on these issues, by considering the social dimension of citizen science projects and guaranteeing diversity and fairness in projects among different stakeholders. On another level, the COST Action has bridged the divide between practitioners and those with a more theoretical approach. It has created spaces for listening to each other, thus increasing reflection on the practice while influencing theory based on existing experiences. Lastly, COST Action activities have allowed participants to find spontaneous ways to contribute to citizen science.

For early career researchers, the COST Action has been a great opportunity to become fully immersed in the citizen science universe facilitating horizontal-level discussions. This is often difficult to achieve coming from institutions or countries where citizen science is not well established and can be viewed with some scepticism. Additionally, working on tasks such as the current book, a lesson in collective writing and editing, has been challenging for early career researchers but allowed them to develop new skills.

Furthermore, most of the COST Action meetings included representatives from non-governmental organisations (NGOs) and civil society organisations working in the field of citizen science. This has allowed the COST Action to have a holistic perspective of citizen science practices which extends far beyond academia. This is crucial as it lies at the heart of what citizen science is. Citizen science is not only about scholars from academia in disciplinary fields; it is also about transdisciplinary cooperation across various scientific disciplines and across the boundaries of various sectors of society (private entities, public entities, NGOs, and non-formal entities). Such a *mixed economy of citizen science* – cooperation, collaboration, and exchange across stakeholders and sectors – is always a challenge (cf. Irwin 1995; Powell 2007).

The COST Action has stimulated the development of the science of citizen science from the perspectives of the *quadruple* (science, policy, civil society, economy) and *quintuple innovation* (plus environment) *helix framework* (see Carayannis and Campbell 2010; Carayannis and Rakhmatullin 2014) and *open innovation 2.0* which entails integrated collaboration and co-created shared value (see Curley 2016; Curley and Salmelin 2018). Due to the high level of heterogeneity, every occasion to meet and discuss increased innovation and creativity.

## The Book

The book was planned from the inception of the COST Action. More than a year before writing the book, the co-chairs started brainstorming with a small group of COST Action members about the possible contents and the target audiences. Together, we also discussed how to include the COST Action’s achievements. From this discussion, the structure naturally emerged, with three main parts: Citizen Science as Science, Citizen Science and Society, and Citizen Science in Practice. Based on the list of COST Action activities being organised, a tentative list of chapters along with an initial list of related authors was relatively easy to establish. The final configuration of the list of authors was left open, and anyone in the citizen science community was invited to join in the writing of any of the planned chapters.

Given the emerging dynamics, writing or acting as editor for the book meant being part of a European citizen science community. The final list of chapters includes more than 100 authors from 23 countries. Collaboration with the editorial team has also been very productive. We divided tasks so that two editors closely supervised each part of the book. The evolution of each part was shared with the whole editorial team periodically to discuss the content as whole.

We also involved all the authors in the editorial process. They were asked to peer review other chapters as part of the quality control process. During a meeting halfway through the writing process, authors and editors discussed the chapters and the final shape of the book. We aimed to make the authors feel like part of the publication team, and they were able to follow the overall editorial process in an integrative and transparent way. The majority of the chapters are co-written by a group of authors that had not previously written together. The diversity of authors in each chapter is another important factor. In this way, the book has organically developed a high degree of interdisciplinarity and inclusiveness in its contents, which is an important characteristic of the citizen science spirit in Europe. With collective and diverse knowledge, we have covered important issues that a person who is new to the field of citizen science would need to know.

Therefore, the book functions as a handbook rather than as an encyclopaedia or an exhaustive collection of citizen science examples. The book aims to represent the current state of the art of the field. However, this does not avoid the fact that chapters may need to be updated due to the rapid evolution of citizen science practices. We also believe that the book succeeds in combining both theoretical and methodological chapters, reflecting the practice of citizen science. There is a swift transition from the focus on theoretical descriptions and analysis to the practical specifications, tools, and guidelines that can be of substantial value, not only for academic communities but also for citizen groups, civil society organisations, and policymakers wanting to embrace citizen science practices.

Academics who are new to the field of citizen science will find the book interesting since it can provide a solid basis for discovering insights and discourses. The term citizen science itself may at first seem quite straightforward, but behind its participatory spirit lie different interpretations of the active presence of citizens in scientific research. The ambiguities and differences in its definition may seem counterproductive to the consolidation of the citizen science field. However, the fluidity and dynamism of the concept not only strengthens citizen science but also describes the heterogeneity and diversity of the community. The newcomer is also able to become acquainted with the theoretical perspectives of citizen science, including research topics where citizen science can be implemented and different aspects of citizen science practice. Illustrated with case studies, the book provides guidance on how to organise a new citizen science project while stressing the key multidisciplinary nature of citizen science practices.

In fact, the last part of the book, Citizen Science in Practice, is targeted at project managers. The chapters cover the practical aspects that need to be considered and lead the practitioner through guidelines for establishing a new project and outline key aspects, such as ethics and data management. Project managers will appreciate advice on standardised ways to disseminate citizen science projects and will learn strategies to make projects more sustainable and interoperable with other projects. The advice also includes discussion of the design of apps and platforms to support citizen science project goals. Key insights on the effort required to develop and maintain apps and platforms are balanced with the ability to use off-the-shelf solutions.

We also aimed to make this book relevant for policymakers, policy officers, and public managers who work in various institutional environments at all levels: local, regional, national, European, and international. They can further reflect on what is needed to move forwards by transforming citizen science knowledge into dedicated and focused policy actions. In the book, entities supporting science can find practical tips for government employees responsible for collaboration with academia and the public, including the dissemination of results of scientific activities. The book can also help experts working at the regional and local levels who are responsible for direct cooperation with civil society organisations, by illustrating key aspects needed to organise and implement citizen science activities.

Ideas and recommendations provided can also be easily adapted to the specific needs and conditions of public programmes and funding schemes, as well as legislation and associated regulations related to the participatory spirit of citizen science practices. From the perspective of developing such programmes, the book analyses cross-cutting issues in citizen science practices, such as ethics, gender dimensions, and the management of intellectual property, as well as digital platforms and data management. Local, regional, national, and international policymakers can find guidance to support citizen science and to ensure project quality. From a broader perspective, practitioners will also find the evaluation framework invaluable. The evaluation covers scientific, participant, socioecological, and economic dimensions.

Civil society organisations will also find the book insightful. Their role in citizen science is examined in the Citizen Science and Society part. There is much work to be done to connect their mission to scientific activity. The roles of each of the actors and their rationale for engaging in citizen science are discussed. Civil society can appreciate and reflect upon key agents of transformative research, which in some cases might be framed within *citizen social science*. Citizen social science includes concerned persons or groups who are typically excluded from research processes. Prominent examples are AIDS treatment activists and movements; patients’ associations introducing social dimensions in mental health care; and environmental justice movement organisations, which focus on the vulnerability of specific social groups. Organisations hoping to identify the problems affecting our environment and our societies might find this particularly informative. The different audience attributes and project types being presented could inform the design of their own projects.

## Possible Futures of Citizen Science

The writer Mark Twain once said: ‘The future interests me – I’m going to spend the rest of my life there’. We want to close this book by delivering some thoughts about the possible futures of citizen science and the challenges that citizen science will face (Bonney et al. 2014). Despite the risk of getting it completely wrong, and thus being discredited when the future becomes present, this is a necessary exercise to further reflect on the nature of citizen science practices.

### Funding

In the short term, the most important challenge might be funding. With a few exceptions – such as in Austria, Germany, the Netherlands, and the United Kingdom – citizen science is not consolidated in national research programmes. It is true that in other countries, such as Spain, citizen science projects receive funding, but it is constrained to public awareness and science communication funding programmes. These programmes are generally modest and tend to omit the quality of research outputs when evaluating proposals. Unfortunately, on a more strategic level, there will be austerity measures related to the economic and political consequences of the COVID-19 pandemic for several years. In general, but especially in peripheral countries where citizen science is still young, it could be challenging to secure public funding, and this may limit the advancement of citizen science practices. The lack of funding can hamper the quality assurance of citizen science projects and can have knock-on consequences for the multidisciplinary nature of citizen science.

The short-term evolution of citizen science in Europe is strongly shaped by the EU funding programmes managed by the Research Executive Agency of the European Commission. This research funding organisation has invested more money in citizen science projects over the last 3 years. Its funding scheme asks for consortia composed of at least three European countries so that the project unites and aligns efforts at the European level. Horizon Europe will be the ninth European Framework Programme (2021–2027). The scope of the funding calls will have a key role in shaping citizen science in the future, but this programme is still in progress at the time of writing (European Commission 2019, 2020). However, national contacts are anticipating that citizen science and participatory research practices might eventually be included in a transversal manner across the different calls of the work programme instead of having specific calls for citizen science. The citizen science community does not have a consensus view on whether this would be the best strategy to promote the adoption of citizen science principles in a large number of EU research funding calls. The transversal approach has a positive aspect because it recognises citizen science practices within the scientific research world, and this could be a path to becoming mainstream. However, there is a risk of downgrading the ambitions of citizen science if they are instrumentalised and trivialised by the current research establishment. The alternative would be to preserve citizen science in a limited but protected space with topic specific calls.

### Project Management and Organisation

The current COVID-19 pandemic will also affect management and organisational issues in citizen science projects and may have contradictory effects. We face the challenge of organising most participatory activities differently, due to social distancing measures. Trust and social ties around citizen science activities are currently built mostly through direct and physical contact. Therefore, the call for social distancing also means testing new, alternative ways of communication and interaction. Some citizen science projects may provide opportunities to escape feelings of powerlessness. Citizen scientists may contribute to the search for proteins (for instance, Foldit), but also report personal and societal shifts. The situation could be an opportunity to awaken more global citizen science projects, enhance worldwide distributed activities, or show how citizen science can participate in and enrich socially relevant discourses. In any case, the current dynamics and strategies in citizen science will need to be revised and adapted while trying not to exclude specific communities or groups that are not as comfortable in digital or physically distant spaces. The situation could also disincentivise disparate initiatives. This could limit the duplication of citizen science efforts and make them more efficient, coordinated, and distributed across countries and disciplines. The crisis could also be an opportunity for the development and further dissemination of innovative citizen science methods and tools.

The next few years could be a testing time to prove the usefulness and effectiveness of new ways of organising scientific processes and scientific organisations. The academic community could be driven by the socio-economic situation to be more open and receptive to exchanges and collaboration with citizens, public entities, and civil society organisations which want a more adaptive, responsive, and agile science to respond to societal challenges. The next few years could lead to a ‘new enlightenment’ (cf. the Enlightenment 2.0 programme of the European Commission’s Joint Research Centre^2^; Mair et al. 2019). New hybridised research methods and tools will emerge from the collaborative efforts that might be facilitated at the local and regional levels. The need to find cost-effective solutions to gathering data and achieving novel scientific results could favour citizen science practices.

Over the next 20 years, citizen science will have to deal with societal factors that are liable to drastic and unexpected change. For example, labour conditions will be modified, and it is unclear how, and if, volunteering, spare time, and employment will overlap. Also, science in general, and research infrastructures and universities in particular, is rapidly moving towards a more flexible and permeable environment.

Another important aspect is the need to further advance the consolidation of mutual learning spaces for the community of practitioners. ECSA and EU-Citizen.Science are helping with this challenge across Europe. EU-Citizen.Science offers a meeting place for researchers, policymakers, civil society organisations, and individuals. However, there are still many metalevel issues that will need to be deeper considered. The most important challenge might be to deepen the exchange of experiences between countries and cultures. Some other common challenges exist around how to engage with those who are not initially interested in science and how to embrace diversity. These latter efforts are related to a better understanding of the impact of participation on scientific literacy. This in turn is related to the power of citizen contributions in successful scientific projects and the potential of bottom-up approaches and co-creation strategies to develop innovative science.

### Impact

In Germany, for instance, there is a demand for proof of impact in citizen science practices, and we expect to see this expanding to other countries. Once citizen science has matured, there will be a greater need to show how citizen science is improving standard research practices, how citizen science can result in better and more representative data, how participation promotes democratic values and collective decision-making for new policies, and how schools can provide motivation in *science, technology, engineering, and mathematics* (STEM) subjects. These are signs that citizen science is maturing, but this open framework will also require the citizen science community to increase their quality standards in an extremely wide set of aspects, much wider than those demanded in standard scientific research projects.

We, as authors, also take a longer-term perspective of 5 years. We expect a stronger citizen science presence inside the scientific system, but also with a more transformative spirit. Citizen science practices reveal the ongoing challenges of citizen engagement and inclusivity. It will become even more important to address these due to the expected increase in inequalities and socio-economic divides. Citizen science will continue to prove its value by providing appropriate arguments to engage and communicate with each of the different stakeholders. We envision that success will also be linked to better representations of the different strata in our societies. This will be a key challenge for citizen science engagement, and a vital one, if citizen science is to uphold its shared values. For many policymakers and scholars, citizen science methods are still not seen as comparable to traditional statistical sampling methods, such as randomised controlled trials and representative surveys. Citizen science will have to find ways to further show the robustness and, by extension, the validity of their scientific results.

### Technology

Given the recent progress in *data science*, data sharing among participants could become easier and safer due to better digital tools. Also, with the rapid advancement of artificial intelligence, some tasks done today by citizens could also be, at least partially, replaced by algorithms – the concept of participation will in turn need to be reconsidered, especially in contributory projects using *crowd science* strategies. This effect could, in fact, increase the pre-eminence of the co-creation component in citizen science projects, thus providing citizens more opportunities to be engaged in all aspects of the research process. We still do not know what the technological factors will be and which emerging technologies will be implemented in the upcoming years. However, we can already anticipate that mobile phones and their evolved forms will be bundled with a myriad of low-cost sensors relevant for citizen science observations. Mobile phones will become powerful enough to process sensor data on the spot, with the help of artificial intelligence and machine learning computational efficiency. The Internet of Things, distributed computing, cloud computing, and cognitive computing will surely transform the concept of participation when dealing with data interpretation in a citizen science project. A good part of the data analysis could be done in the field in near real time, and citizens could benefit from in situ information provision. In combination with social media, individuals and their backgrounds will also personalise data generation and conversations with volunteers. This effort needs to be carefully balanced with privacy issues and any exacerbation of inequalities and social exclusion. In relation to some contentious topics, such as environmental pollution, the ability to preserve participants’ privacy could become a serious issue in countries where freedoms of speech and of information are not fully respected. Citizen scientists could be prosecuted and even receive death threats if they report sensitive observations.

### Participation

Due to the current trend to intensify hands-on and inquiry-based learning strategies, educational resources linked to citizen science will be even more present. Participatory citizen science tasks will have a stronger learning focus, both within formal and curricular education and informal lifelong learning contexts. We also expect that citizen social science will increase its relevance, with a stronger role for civil society organisations, embracing participatory strategies to strengthen their mission. This could position citizen science as more closely aligned to social and environmental actions. Citizen science would in all likelihood develop more hybrid forms that are less subordinate to academic rules and structure.

The COVID-19 crisis might also affect participants’ willingness to collaborate since citizen priorities could change rapidly during the socio-economic crisis that experts are anticipating. For many people, participation in citizen science projects may no longer be attractive. They may now lack the necessary spare time to undertake the planned tasks. More dedicated analysis about benefits and advantages will be necessary in terms of social, human, cultural, and creative capital. While the natural sciences may still hold a dominant place in the citizen science world, an increase of citizen science projects related to social and health issues might also be anticipated. This will be encompassed by citizens’ growing need to empower themselves in these issues due to the likely increase in socio-economic inequalities, alongside a decrease in public funding for health-care services. Also, based on the strong debates on the use of apps for tracing contacts during the COVID-19 crisis, public opinion may have a higher sensitivity to data privacy and ethics (Council of Europe 2020). These will now need to be considered with even more rigour in citizen science digital platforms.

### Research

The overall quality of citizen science projects will still be pursued, but challenges may no longer be primarily linked to increasing the presence of citizen science practices in academia. Citizen science could then have more opportunities to engage with diverse stakeholders. Scientific research would be less exclusive. Anyone in society could have access to the necessary tools and resources to undertake research. The publication of scientific results will change, seeking transparency, accessibility, flexibility, and even more impact. An educator with his or her own classroom could eventually take leadership of a global project. In relation to specific topics, the first-hand experience of concerned groups or communities will gain relevance. For instance, the involvement of older people in the co-creation processes of scientific research will be fundamental to informing better understanding of population ageing. At a lower level of intensity, further development of remote work and new employment formats will shape more strongly what we understand as citizens’ contributions to citizen science projects.

Environmental issues and the climate change emergency are key global issues and will be aligned to a sense of urgency and need for immediate action, with implications for citizen science projects. *Fake news* and bubbles of information will also be widespread digital phenomena and will deeply influence our societies; citizen science practices could confront them and the polarisation of society by creating a productive dialogue through jointly gathered evidence and data (see Mair et al. 2019). Digital platforms, which today are looking for new ways to understand democracy, could also find in citizen science a perfect partner to enhance empowerment and consensus building. These driving forces will surely modify the current ways of designing citizen science projects, which perhaps will be more related to individual well-being, lifelong learning, and social ties.

## Conclusion

The challenge of transferring and exchanging good practices, as this book aims to do, will always exist in the citizen science community. The transparency and honesty of scientific results is something to be valued. Improving the replicability and scalability of projects will require investment of time. There will always be space to improve the participation of the public and other stakeholders in our diverse societies. If science is about knowledge and satisfying our endless curiosity as humans, citizen science will always represent the desire to make this journey together as a global and diverse society.

## Footnotes

1 COST Actions help to connect research initiatives across Europe and beyond and enable researchers and innovators to grow their ideas in any science and technology field by sharing them with their peers. COST Actions are bottom-up networks with a duration of 4 years that boost research, innovation, and careers.

2 https://ec.europa.eu/jrc/en/enlightenment-research-programme
